# Effect of the COVID-19 pandemic on antibiotic consumption: A systematic review comparing 2019 and 2020 data

**DOI:** 10.3389/fpubh.2022.946077

**Published:** 2022-10-18

**Authors:** Mizuho Fukushige, Nhat-Hoang Ngo, Donny Lukmanto, Shinichi Fukuda, Osamu Ohneda

**Affiliations:** ^1^Faculty of Medicine, University of Tsukuba, Ibaraki, Japan; ^2^Laboratory of Regenerative Medicine and Stem Cell Biology, University of Tsukuba, Ibaraki, Japan; ^3^Laboratory of Advanced Vision Science, University of Tsukuba, Ibaraki, Japan; ^4^Department of Ophthalmology, University of Tsukuba, Ibaraki, Japan

**Keywords:** antibiotic consumption, COVID-19, antibiotic stewardship, systematic review, hospital, community

## Abstract

**Background:**

The coronavirus disease 2019 (COVID-19) pandemic has influenced antibiotic consumption over a long period, with variability in trends among studies. We conducted this systematic review to explore and compare the effect of the pandemic on overall and individual antibiotic consumption in 2020 with that in 2019.

**Methods:**

This systematic literature review was conducted using PubMed, EMBASE, and Web of Science databases. Data on antibiotic consumption in Japan was sourced from the Japan Surveillance of Antimicrobial Consumption.

**Results:**

A total of 1,442 articles and reports were screened, and 16 eligible articles were reviewed. The included studies were conducted in Jordan, Australia, Canada, UK, Japan, Brazil, India, China, and the EU. There was no study from African and Southeast Asian Countries. Overall, antibiotic consumption in the community consistently reduced in 2020. Studies from Australia, Canada, Portugal, Spain, the UK, Japan, and the European Union reported both decreases in overall and selected individual antibiotics consumption. In contrast, hospital-based studies reported both increases and decreases. Hospital-based studies in Lebanon, Spain, Italy, India, and the UK reported an increase in antibiotic consumption in 2020. Studies reporting an interruption of antibiotic stewardship programs during the pandemic also reported increases in antibiotic consumption for hospitalized patients in 2020 compared with that in 2019.

**Conclusion:**

Our results showed a different trend between communities and hospitals in antibiotic consumption during 2020 compared to 2019. The continuity of the antibiotic stewardship program might have influenced the antibiotic consumption trend variability among hospitals in 2020. Alongside this, the lack of information on antibiotic consumption from low-income countries and limited reports from middle-income countries revealed gaps that need to be urgently filled.

## Introduction

Antimicrobial-resistant (AMR) bacterial infections—which cause longer hospital stays and higher treatment costs and mortality rates—have been regarded as one of the biggest global health issues (1, 2). Death caused by or associated with AMR bacterial infection has been reported to be the highest in sub-Saharan African countries (1). The overuse of antibiotics can accelerate the emergence of AMR (2). Thus, a key element in AMR control is antibiotic stewardship programs developed to prevent antibiotic over prescription and overuse (3, 4).

The coronavirus disease (COVID-19) pandemic has impacted many aspects of our daily lives, including economics (5), social healthcare (6), and healthcare services (7, 8). A number of studies have reported an immediate increase in antibiotic use during the first wave of COVID-19 (9–11). This was partly because of 1) the treatment of bacterial coinfection among COVID-19 patients (10, 12) and 2) the extra workload for infectious disease professionals, resulting in the interruption of antibiotic stewardship programs (13). However, following the initial increase in antibiotic consumption, a reduction in antibiotic consumption has also been reported (14). Recent publications on yearly antibiotic consumption in 2020 provided us with an opportunity to conduct this systematic review. The COVID-19 pandemic and its potential effect on antibiotic consumption have been reported (Table 1). However, these potential factors that might affect antibiotic consumption could have an opposite effect. Therefore, this study aimed to review the current reported trend of change in antibiotic consumption from 2019 to 2020 during the COVID-19 pandemic and explore differences in the target population (hospital inpatients and community).

**Table 1:** Summary of potential effect of the COVID-19 pandemic on health systems, society, and personal attitude/knowledge that could influence antibiotic consumption (increase or decrease).

**Target**	**Potential effect of COVID-19 on antibiotic consumption**	**Direction of impact**	**Refs**.
Community	City/country lockdown, school closure, state of emergency	Decrease	(19, 31, 32)
	Reducing people-people contact could reduce the opportunity of infectious disease transmission in society.		
Individual	The hesitation of visiting hospitals		(40)
	- People with mild symptoms avoid visiting hospitals	Decrease	
	- Impatient might have more severe conditions	Increase	
Individual	The general public's increased knowledge about infectious disease transmission and application of preventive measures such as hand washing, wearing masks, and maintaining social distancing.	Decrease	(41)
Hospitals	Interruption or reduction of antibiotic stewardship program owing to the extra workload for health professionals to react to the COVID-19 pandemic	Increase	(13, 14, 25)
Hospitals	Extra enhanced hand hygiene/environmental cleaning	Decrease	(42)

## Materials and methods

### Systematic review

An electronic literature search of the PubMed, Web of Science, and EMBASE databases was conducted. The search terms used were (antibiotic OR antimicrobial) AND consumption AND (COVID-19 OR SARS-CoV-2). The antibiotic consumption in Japan and Japanese hospitals were identified using data from the Japan Surveillance of Antimicrobial Consumption (JSAC) database (https://amrcrc.ncgm.go.jp/surveillance/Surveillance_en.html) (15). The search was completed on the February 8, 2022. Duplicate articles were removed, after which titles and abstracts were reviewed. Non-English articles were included in the study, and non-English articles were translated into English for consideration using DeepL (DeepL.com). The entire procedure of the systematic review was conducted by two independent reviewers: MF and NNH.

A study was considered eligible if it met all the following inclusion criteria: 1) involved human participants; 2) reported annual overall antibiotic consumption in 2019 and 2020; 3) quantified antibiotic consumption at a hospital or hospital department or area or country level; and 4) controlled number of inpatients or admissions when they reported hospital-based consumption. A study was considered ineligible if it met any of the following exclusion criteria: 1) only involved non-human subjects; 2) only reported quantified antibiotic consumption among specially selected study participants based on their health conditions (e.g., COVID-19, HIV, diabetes); 3) indirect measurements to quantify antibiotic consumption (e.g., questionnaire, detection in the wastewater); 4) failed to report the measuring unit of antibiotic consumption; 5) hospital-based study that did not control the number of patients when they reported the quantity of antibiotic consumption; and 6) failed to report the quantity of total antibiotic consumption, 7) case reports, review, opinion, and/or study protocol.

### Antibiotic consumption, prescription, and dispensation

The most accurate index that reflected antibiotic use among the targeted populations was antibiotic consumption. However, some studies used antibiotic prescription or dispensation as a proxy of consumption because of the difficulty in accurately measuring antibiotic consumption at the community level. Therefore, community-based studies reporting antibiotic prescriptions and/or dispensations were also included.

### Study period

The main objective of this study was to explore trends in antibiotic consumption over a long period during the COVID-19 pandemic. Therefore, studies that reported the annual consumption of antibiotics in both 2019 and 2020 were initially considered in this review. In addition, studies that reported a quasi-annual consumption (i.e., antibiotic consumption up to October 2020) were included in this review. Studies that exclusively focused on antibiotic consumption during the first wave of the COVID-19 pandemic were excluded.

### Measurement of change in antibiotic consumption quantity

Several studies reported the statistical significance of the change in the quantity of antibiotic consumption during the study years. In this case, their categorization of the trend of this change was followed, namely an increase, no significant change, or a decrease. In contrast, some studies reported only the total quantity of annual antibiotic consumption without any statistical analysis. In this case, their categorization of the trend was followed, namely a ≥10% change between 2019 and 2020. These studies were classified into increase or decrease categories. The remaining studies were classified as the no-change category.

### Quality assurance of studies

Quality assurance of the studies was conducted using a graded scale. The seven criteria were considered in the graded scale: 1) source of antibiotic consumption data was described; 2) antibiotics considered in the study were reported; 3) study period was reported, 4) pre-COVID-19 pandemic antibiotic consumption quantity was reported; 5) antibiotic consumption quantity during the COVID-19 pandemic (or in 2020) was reported; 6) antibiotic consumption quantity was controlled by the number of inpatients and/or inhabitants; and 7) antibiotic consumption quantity was controlled by the number of days of treatment. The scores of the seven criteria were added, and the quality of the study was categorized as follows: low, 0–2; medium, 3–5; and high, 6–7. Based on these criteria, there were no low-quality studies (Supplementary material 1).

## Results

### Systematic review results

**Figure 1:**
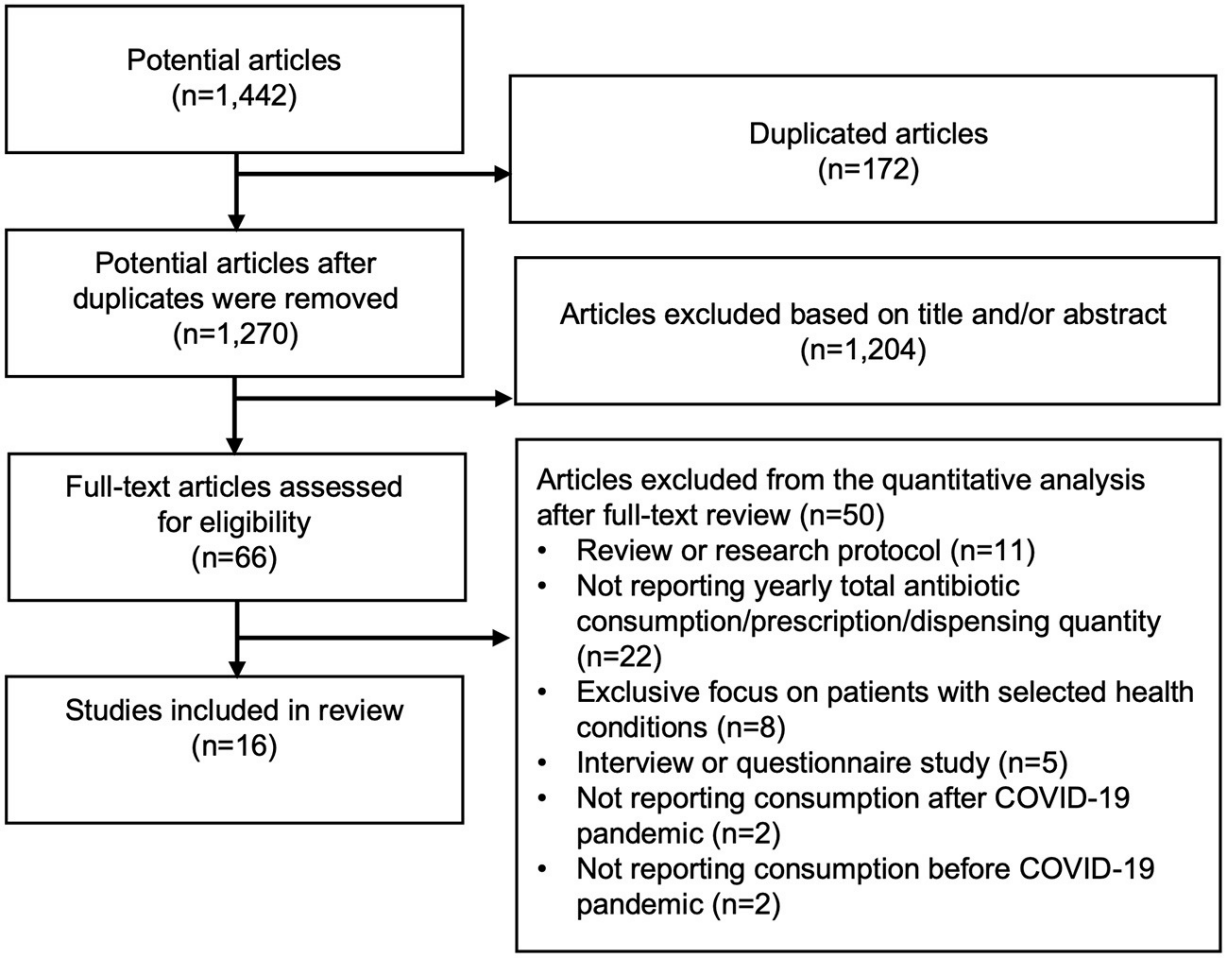
A systematic review flow diagram. Diagram of the number of articles and reports identified and examined at each stage of the review. A total of 16 articles published from 2021 to 2022 met all inclusion criteria and were included in the review.

Through an electronic literature search, 1,441 articles were identified. In addition, one article was identified from the JSAC database. After excluding 172 duplicated articles, 1,270 articles were reviewed and 1,204 articles were excluded by titles and abstracts because they were clearly not relate to our current study. Full text of remaining 66 articles were reviewed, and finally, 16 articles were included in this systematic review (13–28) (Figure 1). Eight studies reported only hospital-based results (13, 14, 23–28), nine reported only community-level antibiotic consumption (14–22), and one [conducted in England (14)] reported antibiotic consumption in both hospitals and communities (Tables 2, 3). Although we did not limit the studies to the year 2020, we could not find any study that reported antibiotic consumption in 2021 through the systematic review.

**Table 2:** Antibiotic consumption trend at hospital levels showing the overall trend and individually reported antibiotics.

**Authors**	**Country**	**Source**	**Overall trend**	**Unit**	**Antibiotics**
Chamieh et al. (24)	Lebanon	Saint George Hospital University Medical Center	Increased by 20%	DDD/1,000 PD	Carbapenem
Grau et al. (25)	Spain	Acute care hospitals affiliated with the VINCat program	Increased *P* < 0.001	DDD/100 PD	Cephalosporins Carbapenems
					Other cephalosporins and penems
					Macrolides
					Penicillins
					Aminoglycoside antibacterials
					Quinolone antibacterials
Macera et al. (13)	Italy	The University Hospital luigi Vanvitelli in Naples	Increased Surgical ward	DDD/100 PD	III/IV Generation Cephalosporins (Surgical)
					Aminopenicillins/BLI (Surgical)
					Metronidazole (Surgical)
					Vancomycin
					Linezolid
					Tigecycline
					Cefazolin
					Carbapenems
					Piperacillin/tazobactam
					Fluoroquinolones (ICU)
					III/IV Generation Cephalosporins (ICU)
Padhan et al. (26)	India	AIIMS Raipur	Increased (*p* < 0.0001)	DDD/100 PD	Piperacilli + Tazobactam
					Ceftriaxone
					Azithromycin
					Paracetamol
					Dexamethasone
					Ciprofloxacin
					Diclofenac
					Hydrocortisone
					Meropenem
					Metronidazole
Andrews et al. (14)	UK	The ePACT2 from the NHS Business Services Authority	Increased by 12%	DDD/1,000 admissions	NA
Silva et al. (27)	Brazil	A tertiary hospital in Rio de Janeiro	Stable (*p* = 0.068)	DDD/BD	Polymyxin B
					Polymyxin E
					Daptomycin
					Ciprofloxacin
					Amikacin
					Linezolid
					Ceftazidime/avibactam
					Ceftozolone/tazobactam
					Tigecycline
					Azithromycin
					Cefepime, Ceftazidime, Ceftriaxone, Cefuroxime
					Ertapenem
					Levofloxacin
					Meropenem
					Moxifloxacin
					Piperacilin/tazobactam
					Teicoplanin
					Vancomycin
					Ampicillin, Ampicillin/sulbactam
					Amoxicillin and clavulanate
Liu et al. (23)	China	A Tertiary teaching hospital in Shanghai	Decreased by >10%	DDD/100 PD	NA
Murgadella-Sancho et al. (28)	Spain	Moises Broggi Hospital	Decreased (*p* = 0.045)	DDD/100 BD	NA

Overall trend and individual antibiotics: text in red represents a significant increase in 2020 compared to 2019 and/or an increase >10%. Similarly, text in #3bc1cd represents a significant decrease and/or a decrease >10%. Text in black represents antibiotics that did not show statistically significant change and/or reported < 10% change.

DDD, defined daily dose; PD, patient days.

**Table 3:** Antibiotic consumption trend at regional or national levels showing the overall trend and individually reported antibiotics.

**Author**	**Country**	**Source**	**Overall trend**	**Unit**	**Antibiotics**
Al-Azzam et al. (16)	Jordan	Jordan food and drug administration (JFDA)	Stable (28.4–>26.8 DDD/1,000 ID)	DDD/1,000 ID	Beta-lactamase resistant penicillin Third-generation cephalosporins
					Fourth-generation cephalosporins
					Carbapenems sulfonamides with trimethoprim
					Macrolides
					Lincosamides
					Combinations of antibacterials
					Tetracyclines
					Other aminoglycosides
					Fluoroquinolones
					Combinations of penicillin
					Second-generation cephalosporins
					Glycopeptide antibacterials
					Other cephalosporins and penems
					Penicillin with extended-spectrum
Gillies et al. (19)	Australia	National claims data Australia	Decreased by 36.4%	Dispensing/1,000 inhabitants	Trimethoprim Flucloxacillin
					Metronidazole
					Cefalexin
					Amoxicillin
					Amoxicillin with clavulanic acid
					Doxycycline
					Roxithromycin
					Clarithromycin
					Phenoxymethylpenicillin
Knight et al. (20)	Canada	National antibiotic dispensing data from IQVIA's CompuScript database	Decreased by >10%	Dispensing/1,000 inhabitants (mean)	NA
Silva et al. (17)	Portugal	The Portuguese National Health System (NHS)	Decreased by >10%	Prescription DDD/1,000 inhabitants	Third-generation cephalosporins
					Fluoroquinolones
					Clarithromycin
Rojas-Garcia and Antonanzas (21)	Spain	The Department of Pharmaceutical and Health Products of La Rioja	Decreased by >10%	Prescription DDD/1,000 inhabitants	Doxycycline
					Amoxicillin and beta-lactamase inhibitor Amoxicillin
					Cefuroxime
					Azithromycin
					Levofloxacin
Nicieza García et al. (18)	Spain	The Health Service of the Principality of Asturias	Decrease by 24% (*p* < 0.001)	DDD/1,000 insured adult population	NA
Andrews et al. (14)	UK	The ePACT2 from the NHS Business Services Authority	Decreased by >10%	Prescription items/1,000 population	NA
JSAC (15)	Japan	The National Database (NDB) of Health Insurance Claims and Specific Health Checkups of Japan	Decreased by 21%	DDD/1,000 ID	B-lactam antibacterials, Penicillins
					Quinolones
					Macrolides, Lincosamides, and Streptogramins
					Other B-lactam antibacterials
Hogberg et al. (22)	EU (29 countries)	European Surveillance of Antimicrobial Consumption Network (ESAC-Net)	Decreased by >10%	DDD/1,000 ID	Tetracyclines
					Sulfonamides and trimethoprim
					Beta-lactams, penicillins
					Other beta-lactam antibacterials
					Macrolides, lincosamides, and streptogramins
					Quinolones

Overall trend and individual antibiotics: text in red represents a significant increase in 2020 compared to 2019 and/or an increase >10%. Similarly, text in #3bc1cd represents a significant decrease and/or a decrease >10%. Text in black represents antibiotics that did not show statistically significant change and/or reported < 10% change.

DDD, defined daily dose; ID, inhabitants per day.

These hospital-based studies were conducted in Lebanon, Spain, Italy, India, Brazil, China, and the UK (13, 14, 23–28). Community antibiotic consumption studies were conducted in Jordan, Australia, Canada, Portugal, Spain, the UK, Japan, and the EU (14–22). A study in the EU reported the total antibiotic consumption of 29 countries and the antibiotic consumption of 27 individual countries (including Austria, Belgium, Bulgaria, Croatia, Denmark, Estonia, Finland, France, Germany, Greece, Hungary, Iceland, Ireland, Italy, Latvia, Lithuania, Luxembourg, Malta, Netherlands, Norway, Poland, Portugal, Romania, Slovakia, Slovenia, Spain, and Sweden) (22). Notably, all community/national-level studies reported a decrease in overall antibiotic consumption in 2020 compared with that in 2019 (before the COVID-19 pandemic). All community-based studies, except one conducted in Jordan (16), reported that the individual or group antibiotic consumption decreased in addition to the reduction in overall antibiotic consumption. In contrast, hospital-based studies have reported an overall increase and decrease in antibiotic consumption in 2020.

Similarly, Andrews et al. reported a decrease in antibiotic prescriptions in the community in 2020 and an increase in hospital admissions in England (14). They also reported a decrease of more than 10% in total antibiotic consumption at the community level from January to October 2020 compared with that in the same months in 2019. However, the consumption at hospitals in DDDs/1,000 admissions increased by 12% during the same study period in 2020 compared with that in 2019.

### Reported interruption of antibiotic stewardship program in 2020

The interruption or reduction of antibiotic stewardship programs during the COVID-19 pandemic has been reported by Grau et al. (25), Macera et al. (25), Andrews et al. (14), and Murgadella-Sancho et al. (28). These studies, except for Murgadella-Sancho et al., reported an increase in antibiotic consumption in 2020. Only one study from China reported that the continuous implementation of antibiotic stewardship programs since 2019 throughout 2020 resulted in the reduction of overall antibiotic consumption in 2020 (23).

### Economic levels of countries where a study was conducted

The economies of countries were categorized according to the World Bank country and lending groups (29). Hospital-based studies have been conducted in high-income economies (Spain, Italy, and the UK), upper-middle-income economies (Lebanon and China), and lower-middle-income economies (India and Brazil) (13, 14, 23–28). All community-based studies were conducted in high-income countries, except Jordan, an upper-middle-income economy (14–22). Neither hospital nor community-based studies have been conducted in low-income economies.

## Discussion

Our systematic review results revealed a consistent decrease in community antibiotic consumption (14–22). In contrast, more studies reported an increase in overall hospital antibiotic consumption in 2020 compared with that in 2019 (13, 14, 23–28). Multiple hospital-based studies reported the interruption of antibiotic stewardship programs in 2020 because of the allocation of infection control specialists for COVID-19, which resulted in increased overall antibiotic consumption (13, 14, 25). However, only one study reported that the continuous antibiotic stewardship program that was started from 2019 throughout 2020, resulted in a decrease in antibiotic consumption in 2020, despite a transient increase during the first wave of COVID-19 (23). Furthermore, one study from Spain reported a continuous antibiotic stewardship program during the pandemic with some amendments to react to the change in human resource allocation (28). The study reported a decrease in antibiotic consumption in 2020, showing the possibility of continuous antibiotic consumption control while fighting against the pandemic. This review highlights the importance of antibiotic stewardship programs even during the COVID-19 pandemic.

### Decrease in antibiotic consumption in the community

All the studies identified in our systematic review reported a decrease in antibiotic consumption at the national level (14–22). The decrease in antibiotic consumption in the community could reflect 1) a decrease in antibiotic use for respiratory infection, which reflects the effectiveness of personal infection protection efforts of individuals in the community; 2) the hesitancy of visiting hospitals when people had no/light/mild symptoms owing to the fear of contracting COVID-19 (30); 3) reduced social activities observed through national infection control in many countries, e.g., city or country lockdown, which decreases the likelihood of getting an infection when amidst other people (31, 32); and 4) increased awareness and infection prevention activities among the public (e.g., enhanced hand hygiene, universal wearing of masks, and social distancing).

Increased public awareness regarding infectious disease control was a positive side effect of the COVID-19 pandemic. However, regardless of the benefits of strict regulations such as city/country lockdown and state of emergency in COVID-19 control, the pandemic has affected the economy (33) and mental health (34) of the nation. Therefore, such strict infection control measures cannot last for a long time. Antibiotic consumption must be continuously and carefully monitored once social restrictions are lifted.

### Decrease/increase in antibiotic consumption in hospitals

Contrary to the consistent decrease in overall antibiotic consumption in the community, there was a different trend in antibiotic consumption in hospitals (13, 14, 23–28). An increase was reported in hospitals in Spain, Italy, India, and the UK. However, a decrease was reported in hospitals in China and Spain. This variability in antibiotic consumption in 2020 compared with that in 2019 could be caused by 1) the levels of antibiotic stewardship program implementation during the COVID-19 pandemic; 2) the reluctance of people to visit hospitals during the COVID-19 pandemic (30) hence, those hospitalized were likely to have more severe symptoms than that noted in the regular year; and 3) the use of antibiotics to treat COVID-19 patients (12, 35).

One study from Spain reported an interrupted antibiotic stewardship program in 66 acute care hospitals and increased antibiotic consumption (25). In contrast, another study from Spain reported that a continuous antibiotic stewardship program with necessary amendment to the reaction to the COVID-19 pandemic caused a decrease in antibiotic consumption (28). Similarly, Liu et al. also reported that the implementation of an antibiotic stewardship program from 2019 to 2020 resulted in a successful reduction of overall antibiotic consumption by 2020 (23). These studies highlight the benefit of a continuous antibiotic stewardship program even with some amendments.

### Lack of reports from low-income countries

Our review results showed that the studies were mostly published in high-income countries. Only one community-based study was reported from a middle-income country (Jordan) (16) and three hospital-based studies from India (26), Brazil (27), and China (23). Furthermore, no studies have been conducted in low-income countries. Sub-Saharan Africa has been reported to have the highest rate of death associated with AMR (1). Moreover, the antibiotic market is expected to grow rapidly in middle- and low-income countries (36). An interview-based study in Nigeria reported up to a 6-fold increase in antibiotic use among local pharmacists during the first wave of COVID-19 in 2020 (37). There have been reports concerning the inappropriate and increased use of antibiotics during the COVID-19 pandemic in low- and middle-income countries, such as Zimbabwe (38), Malawi, and Uganda (39). This includes antibiotics used in the hospital and at home (over-the-counter drugs without a prescription). Although these reports are highly concerning, we could not identify any quantitative studies or governmental reports that contained the quantity of antibiotics consumed in these countries. More information on antibiotic consumption in middle- and low-income countries is urgently needed.

### Limitations

This review excluded studies that exclusively focused on patients with specific comorbidities, including COVID-19. However, the magnitude of the effect of antibiotic consumption on COVID-19 patients could not be distinguished from hospital- or department-level antibiotic consumption, even though this effects the overall antibiotic consumption trend. National-level studies (e.g., national antibiotic consumption in Japan) included both outpatient and inpatient antibiotic consumption. Thus, although the main interest was antibiotic use in the community, this report included inpatient antibiotic use. Because of the type of data, we could not distinguish between these data in some community- and national-level studies. In addition, the variability of the size of the targeted population could not be directly considered in the current study.

## Conclusion

During the COVID-19 pandemic, all community- and national-level studies reported an overall decrease in antibiotic consumption. This could be because of the reduction in infectious disease cases induced by social and individual control of infection. In contrast, hospital inpatients' antibiotic consumption was reported to decrease or increase depending on the hospital. This could be because of the increase in the ratio of more severe patients during the pandemic and the interruption of antibiotic stewardship programs. Thus, hospitals that reported an interruption of antibiotic stewardship programs in 2020 also reported an increase in antibiotic consumption. Our results highlight the importance of continuing antibiotic stewardship programs even while implementing amendments to manage to the pandemic. In addition, the review showed a research output gap between low- and middle-income countries. More studies evaluating the effect of the COVID-19 pandemic on antibiotic consumption should be conducted in these countries.

## Data availability statement

The original contributions presented in the study are included in the article/Supplementary material, further inquiries can be directed to the corresponding author.

## Author contributions

MF, DL, and OO: initial conception and design of the study. MF and N-HN: systematic review and data extraction. MF, N-HN, DL, SF, and OO: contribution to drafting manuscript editing/reviewing. All authors approved the final version of the manuscript.

## Funding

MF was supported by the Japan Agency for Medical Research and Development (project code: 22fk0108630h0001) and the University of Tsukuba's review paper-editing project. The funders do not play a role in this review results.

## Conflict of interest

The authors declare that the research was conducted in the absence of any commercial or financial relationships that could be construed as a potential conflict of interest.

## Publisher's note

All claims expressed in this article are solely those of the authors and do not necessarily represent those of their affiliated organizations, or those of the publisher, the editors and the reviewers. Any product that may be evaluated in this article, or claim that may be made by its manufacturer, is not guaranteed or endorsed by the publisher.
