# Supplementary material for: Effect of the COVID-19 pandemic on antibiotic consumption: A systematic review comparing 2019 and 2020 data
Source: Front Public Health. 2022 Oct 18;10:946077. doi: 10.3389/fpubh.2022.946077 (PMC9623150; doi:10.3389/fpubh.2022.946077)
Supplement: Supplementary file 1 [file Table_1.docx]

**Supplemental 1. Quality assurance of studies.**

When a study met and/or reported each criterion a score of one was given. A study categorized “high quality” when a total score was 6-7, “medium” with 3-5, and “low” with 0-2.

1. source of antibiotic consumption data was described
2. antibiotics considered in the study were reported
3. study period was reported
4. pre-COVID-19 pandemic antibiotic consumption quantity was reported
5. antibiotic consumption quantity during the COVID-19 pandemic (or in 2020) was reported
6. antibiotic consumption quantity was controlled by the number of inpatients and/or inhabitants
7. antibiotic consumption quantity was controlled by the number of days of treatment

| **Author** | **1)** | **2)** | **3)** | **4)** | **5)** | **6)** | **7)** | **Total** | **Quality** |
| --- | --- | --- | --- | --- | --- | --- | --- | --- | --- |
| Chamieh A 2021 | 1 | 1 | 1 | 1 | 1 | 1 | 1 | 7 | High |
| Grau S 2021 | 1 | 1 | 1 | 1 | 1 | 1 | 1 | 7 | High |
| Macera M 2021 | 1 | 1 | 1 | 0 | 0 | 1 | 1 | 5 | Medium |
| Padhan S 2021 | 1 | 1 | 1 | 1 | 1 | 1 | 1 | 7 | High |
| Andrews A 2021 | 1 | 0 | 1 | 1 | 1 | 1 | 0 | 5 | Medium |
| Silva ARO 2021 | 1 | 1 | 1 | 1 | 1 | 1 | 1 | 7 | High |
| Liu XY 2021 | 1 | 0 | 1 | 1 | 1 | 1 | 1 | 6 | High |
| Murgadella-Sancho AA 2021 | 1 | 0 | 1 | 1 | 1 | 1 | 1 | 6 | High |
| Al-Azzam S 2021 | 1 | 1 | 1 | 1 | 1 | 1 | 1 | 7 | High |
| Gillies MB 2021 | 1 | 1 | 1 | 0 | 0 | 1 | 0 | 4 | Medium |
| Knight BD 2022 | 1 | 0 | 1 | 0 | 0 | 1 | 0 | 3 | Medium |
| Silva TM 2021 | 1 | 1 | 1 | 0 | 0 | 1 | 0 | 4 | Medium |
| Rojas-Garcia P 2021 | 1 | 1 | 1 | 0 | 0 | 1 | 0 | 4 | Medium |
| Nicieza García ML 2022 | 1 | 0 | 1 | 1 | 1 | 1 | 0 | 5 | Medium |
| JSAC 2022 | 1 | 1 | 1 | 1 | 1 | 1 | 1 | 7 | High |
| Hogberg LD 2021 | 1 | 1 | 1 | 1 | 1 | 1 | 1 | 7 | High |
